# Effective hemostasis with a self-assembling peptide hemostatic gel to manage leaky hemorrhage at the ulcer closure site after gastric endoscopic submucosal dissection

**DOI:** 10.1055/a-2291-9050

**Published:** 2024-04-09

**Authors:** Ryohei Maruoka, Mitsuru Esaki, Yosuke Minoda, Yoshihiro Otsuka, Kazuhiro Haraguchi, Haruei Ogino, Eikichi Ihara

**Affiliations:** 1 91356 Department of Gastroenterology, Harasanshin Hospital, Harasanshin Hospital, Fukuoka, Japan; 2 12923 Department of Medicine and Bioregulatory Science, Graduate School of Medical Sciences, Kyushu University, Fukuoka, Japan

Intraoperative bleeding is a major complication of endoscopic submucosal dissection (ESD), presenting challenges in identifying the bleeding source and achieving effective hemostasis. Recently, a self-assembling peptide hemostatic gel (PuraStat; 3D Matrix, Tokyo, Japan) was developed as a hemostatic agent that reacts with blood or body fluids to form a hydrogel that coats the bleeding surface 1 2 3 . This process results in the occlusion of ruptured vessels, halting bleeding by inducing blood coagulation. Here, we report the successful use of a hemostatic gel in controlling leaky bleeding at a fully closed ESD site where the specific bleeding point could not be identified ( Fig. 1 ; Video 1 ).

**Fig. 1:**
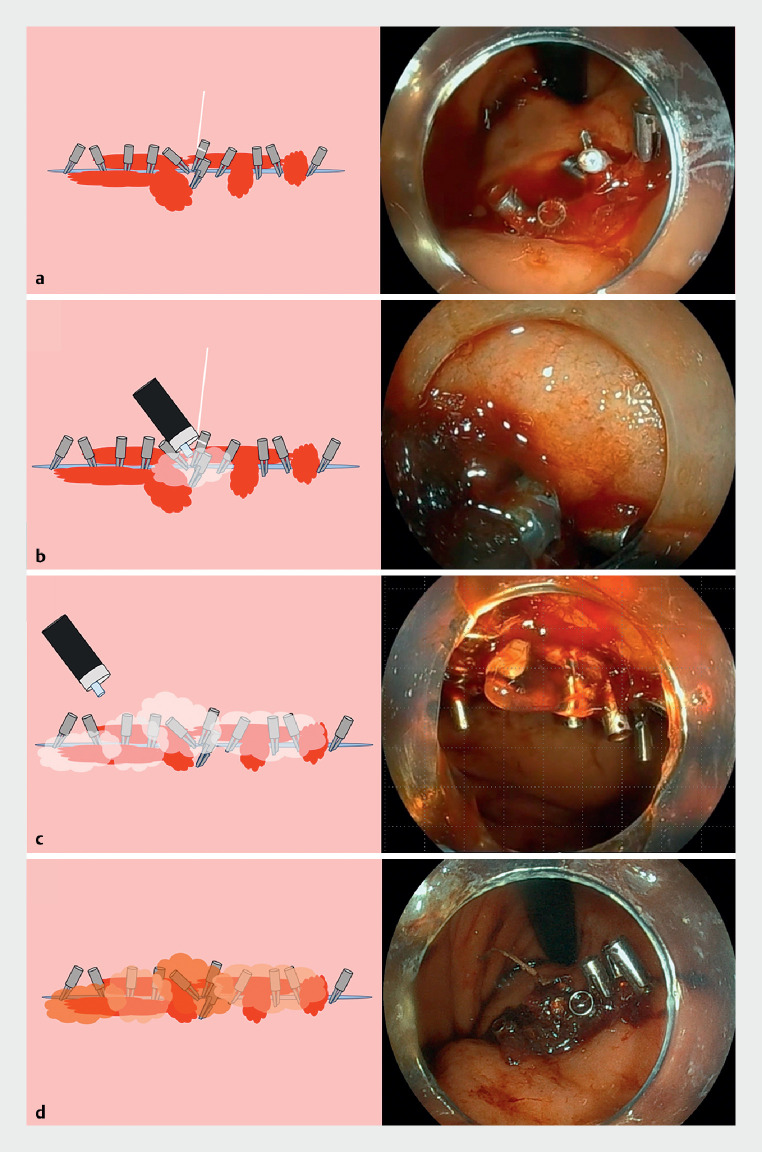
Endoscopic images showing hemostasis using a self-assembling peptide hemostatic gel. **a** Leaky bleeding observed at the ulcer site during the endoscopic closure process. **b** Injection of hemostatic gel into the closed ulcer through the gap between the clips, using a catheter connected to a syringe. **c** Complete coverage of the closure site with additional gel. **d** Successful hemostasis achieved after a short observation period.

Effective hemostasis with a self-assembling peptide hemostatic gel for leaky hemorrhage at the closure site of an ulcer after gastric endoscopic submucosal dissection.Video 1

A 76-year-old man underwent ESD for an early gastric neoplasm measuring 8 mm on the lesser curvature of the gastric body. Although the ESD was successfully completed with en bloc resection, the muscular layer of the post-ESD ulcer was partially injured. To prevent delayed perforation, endoscopic clip closure with thread assistance was applied to the ulcer 4 . However, leaky bleeding occurred at the ulcer site during endoscopic closure, and despite complete closure of the post-ESD ulcer, leaky bleeding from the site persisted. The difficulty in identifying the bleeding point made it difficult to achieve hemostasis using additional clips or hemostatic forceps. To avoid allowing the ulcer to re-open when clips were removed, a self-assembling peptide hemostatic gel was applied to achieve hemostasis. The hemostatic gel was injected into the closed ulcer through the gap between the clips using a catheter connected to a syringe, and the closure site was completely covered with additional gel. Complete hemostasis was achieved after a short observation period. The patient was later discharged without any additional bleeding episodes. The hemostatic gel has proven to be effective in controlling bleeding at the ulcer closure site after gastric ESD, particularly in situations where the specific bleeding point cannot be identified and directly accessed.

Endoscopy_UCTN_Code_CPL_1AH_2AC

## Notes

Endoscopy E-Videos https://eref.thieme.de/e-videos
*E-Videos* is an open access online section of the journal *Endoscopy* , reporting on interesting cases and new techniques in gastroenterological endoscopy. All papers include a high-quality video and are published with a Creative Commons CC-BY license. Endoscopy E-Videos qualify for HINARI discounts and waivers and eligibility is automatically checked during the submission process. We grant 100% waivers to articles whose corresponding authors are based in Group A countries and 50% waivers to those who are based in Group B countries as classified by Research4Life (see: https://www.research4life.org/access/eligibility/ ). This section has its own submission website at https://mc.manuscriptcentral.com/e-videos .
